# Exploring heterologous prime-boost vaccination approaches to enhance influenza control in pigs

**DOI:** 10.1186/s13567-020-00810-z

**Published:** 2020-07-09

**Authors:** Chong Li, Marie R. Culhane, Maxim Cheeran, Lucina Galina Pantoja, Micah L. Jansen, Deborah Amodie, Martha A. Mellencamp, Montserrat Torremorell

**Affiliations:** 1 College of Veterinary Medicine, University of Minnesota, St. Paul, MN 55108 USA; 2 Zoetis, Parsippany, NJ 07054 USA

**Keywords:** Influenza A virus, Heterologous prime-boost vaccination, Disease control, Pig

## Abstract

Influenza A viruses evolve rapidly to escape host immunity. In swine, this viral evolution has resulted in the emergence of multiple H1 and H3 influenza A virus (IAV) lineages in the United States (US) pig populations. The heterologous prime-boost vaccination strategy is a promising way to deal with diverse IAV infection in multiple animal models. However, whether or not this vaccination strategy is applicable to US swine to impart immunity against infection from North American strains of IAV is still unknown. We performed a vaccination-challenge study to evaluate the protective efficacy of using multivalent inactivated vaccine and/or a live attenuated IAV vaccine (LAIV) in pigs following multiple prime-boost vaccination protocols against a simultaneous H1N1 and H3N2 IAV infection. Our data show that pigs in the heterologous prime-boost vaccination group had more favorable outcomes consistent with a better response against virus challenge than non-vaccinated pigs. Additionally, delivering a multivalent heterologous inactivated vaccine boost to pigs following a single LAIV administration was also beneficial. We concluded the heterologous prime boost vaccination strategy may potentiate responses to suboptimal immunogens and holds the potential applicability to control IAV in the North American swine industry. However, more studies are needed to validate the application of this vaccination approach under field conditions.

## Introduction

Influenza A viruses are important zoonotic pathogens and one of the most prevalent causes of respiratory disease. Swine influenza A virus (IAV) causes respiratory disease in pigs worldwide and is considered a significant player in the porcine respiratory disease complex together with other viruses and bacteria such as porcine reproductive and respiratory syndrome virus (PRRSV) and *Mycoplasma hyopneumoniae* (*M. hyopneumoniae*) [1]. Influenza causes important economic losses in the swine industry with estimates ranging between $3 and $10/pig [2]. H1N1, H3N2, and H1N2 are the major subtypes of IAV found in pigs around the world and there is significant genetic diversity within those subtypes [3].

Influenza A virus is an enveloped RNA virus with a genome consisting of 8 negative-sense RNA segments. Its genetic diversity is driven mostly by two mechanisms: antigenic drift which is the result of mutations in antigenic sites due to the poor proofreading ability of the RNA polymerase, and antigenic shift, or reassortment, which is the exchange of gene segments between distinct viruses resulting in new strains with a gene combination distinct from the parental strains. These viral evolution mechanisms are responsible for the emergence of multiple novel distinct H1 and H3 IAV lineages in pigs during the last 20 years [4]. Between 2009 and 2016, 74 genome patterns were documented for the H1 subtype from US swine alone [5]. The high diversity of IAV makes control of the disease using vaccination difficult. The differences of prevailing lineages between different continents and regions require the IAV vaccines for swine to be produced locally and contain distinct strains for each region.

Currently, vaccination is the primary measure to control IAV infection in pigs. The success of vaccination control measures is largely attributed to the ability of the vaccine to stimulate the host immune system and elicit high levels of humoral and cell meditated immune response. The protection imparted by the humoral immune response comes not only through the secretion of anti-hemagglutinin antibodies that neutralize the virus but also from the production of antibodies that stimulate complement system and subsequent antibody-dependent cellular cytotoxicity (ADCC) which further inhibits virus replication [6]. Cellular immunity also plays a vital role in virus elimination by activating cytotoxic T cells and macrophages to lyse infected cells and destroy ingested microbes [7]. Even though much progress has been made to improve the efficacy of recombinant subunit, vector and DNA vaccines against IAV and these have promising results as demonstrated in experimental studies, whole-cell inactivated vaccines (WIV) remain the most commonly used licensed vaccines to control IAV in pigs. WIV are widely used in sows and gilts to induce serum antibodies against the viral hemagglutinin and enhance transfer of passive immunity to newborn piglets. More than 60 percent of large breeding herds in the U.S vaccinated gilts against IAV before or at entry into the herds by using commercial or autogenous WIV [8]. Furthermore, over one-half of large breeding herds vaccinated sows during the last 4 weeks of gestation [8]. In addition, a live attenuated influenza vaccine (LAIV) became commercially available in the US in 2018 [9]. This vaccine, a multivalent LAIV with an NS1 truncated protein, has been purported to elicit both humoral and cell-mediated immune responses in pigs 1 day of age and older [10]. However, there is less overall information regarding the use of LAIV. LAIV are usually administrated intranasally to piglets to bypass or minimize the limiting effect of maternal antibodies.

Due to antigenic drift and shift, vaccines for IAV need to be updated on a regular basis to provide sufficient protection. However, updating the vaccine requires significant time to determine the vaccine composition, testing, distribution, and administration [1]. The delayed time, almost 8 months for human vaccines, is enough for new strains to emerge and undergo antigenic drift and shift, which renders the immunization too late to have substantial impact on the next influenza outbreak [11]. In the case of vaccines for animals, this time may be even longer [12]. Moreover, there is no systematic worldwide surveillance program for IAV in pigs and IAV vaccines for swine are updated far less frequently than humans [13]. Therefore, it is much harder to develop vaccines that incorporate strains that are appropriately matched with the circulating strains in a farm or region and provide complete protection against IAV for pigs. For these reasons, broadening the immune response is key to providing more complete protection and one way to do this is to adopt a heterologous prime-boost vaccination strategy.

Heterologous prime-boost vaccination refers to the delivery of antigens with overlapping nucleotides or same antigenic inserts expressed by different vectors or delivery systems for primary and boost vaccination [14, 15]. Previous research with H5N1 influenza virus in poultry, H1N1 in mice, and H3N2 in ferrets has resulted in the desired broad and long-lasting immune response using the prime-boost approach [16–18]. The benefit of heterologous prime-boost vaccination has also been evaluated in controlled experiments with pigs and the desired broader protection against H3N2 IAV was demonstrated [19]. Whether the heterologous prime-boost strategy is applicable to US swine infected with North American strains of IAV using currently available multivalent IAV vaccines for pigs is still unknown. Therefore, we evaluated the protective efficacy of heterologous prime-boost vaccination with WIV and LAIV vaccines in a swine model against H1 and H3 IAV co-infection. The outcomes of this study may be used to develop better vaccination protocols and new control strategies for IAV in pigs.

## Materials and methods

### Experimental design and pig allocation

**Figure 1:**
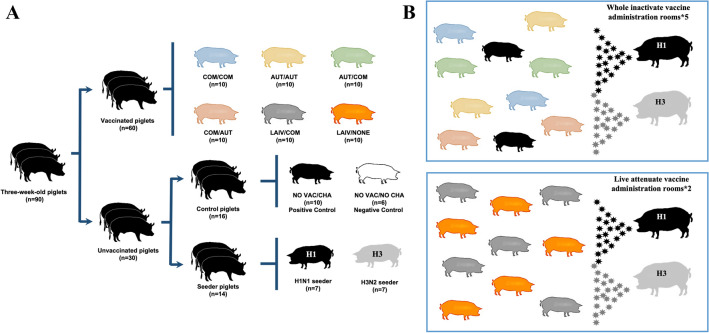
**Diagram showing the experimental design and the pig allocation for each group. A** Distribution of pigs in each treatment group. Pigs from different treatment groups are shown with disparate colors. The total number of pigs distributed in each treatment group (n) is indicated below the pig icons. **B** Distribution of vaccinated and seeder pigs in each room. Colors representing pigs from different treatment groups correspond to colors used in **A**. Top panel indicates distribution of whole inactivated vaccine groups and bottom panel indicates the distribution of live attenuated vaccine groups. The total number of rooms used for housing pigs which received the whole inactivate or live attenuate vaccine administration (*) are also indicated. The six pigs from control group NO VAC/NO CHA were housed in a separate single room which is not shown in this figure.

This project consisted of two separate studies (Table 1). The first study included only pigs vaccinated with commercial (COM) or autogenous (AUT) WIV and had five different treatment groups: COM/COM, AUT/AUT, AUT/COM, COM/AUT and no vaccination but challenged (NO VAC/CHA). The second study included pigs vaccinated with the LAIV and two treatment groups: LAIV/COM and LAIV/NONE (NONE = no boost vaccination). A combined total of ninety, 3-week-old pigs were enrolled in the project. Pigs originated from a farm seronegative to IAV, PRRSV and *M. hyopneumoniae* and the farm was monitored for these pathogens regularly. All pigs tested negative for antibodies against the IAV nucleoprotein (HerdChek, IDEXX ELISA) and negative for IAV RNA in nasal swabs using a matrix gene real-time RT-PCR (RRT-PCR) [20] upon arrival to the University of Minnesota BSL-2 animal isolation units. Pigs were randomly allocated into 8 treatment groups as depicted in Table 1 and Figure 1A. A microchip was implanted intramuscularly in the neck of each pig (LifeChip^®^, Destron Fearing, South Saint Paul, MN) to monitor body temperature. Sixty of the pigs were vaccinated against influenza using different prime-boost vaccination protocols at 3 and/or 6 weeks of age. The vaccines were administered according to their labels. The COM and AUT vaccines were administrated intramuscularly (IM) with 2 ml per dose, while, the LAIV was administrated as a single 1 ml dose intranasally (IN) for each pig. Both, sixteen control pigs (include NO VAC/CHA and NO VAC/NO CHA groups) and fourteen seeder pigs received two administrations of a saline solution intramuscularly at 3 and 6 weeks of age. The seeder pigs were challenged with either an H1 or H3 IAV at 8 weeks of age and served as infection sources to the vaccinated pigs and NO VAC/CHA pigs. Each seeder pig was inoculated intratracheally and intranasally with a 2 ml dose of 1 × 10^6 TCID_50_/mL challenge virus (1 mL intratracheally and 1 mL intranasally). When seeder pigs were confirmed IAV positive in their nasal secretions by RRT-PCR, two seeder pigs (one H1 seeder and one H3 seeder) were commingled with the pigs in each room. There were five rooms in total for the Study 1 with each room containing ten contact pigs (two from each WIV treatment group), and two seeder pigs for a total of 12 pigs per room (Figure 1B). Similarly, the two rooms in the second study had twelve total pigs per room with five pigs from LAIV/COM, five pigs from LAIV/NONE and two seeder pigs (Figure 1C). Six pigs from NO VAC/NO CHA group served as unvaccinated negative controls and were kept in a separate room. Three of the NO VAC/NO CHA pigs were euthanized at 8 weeks of age (0 days post-contact (dpc)) for histopathological evaluation and the remaining of the 3 pigs were euthanized at 9 weeks of age (7 dpc).

**Table 1:** Description of vaccination protocols applied to pigs by treatment groups in the two separate studies.

Study	Description	Group	Vaccination		Route	Challenge	Necropsy
			Prime	Boost			
1	Whole inactivated vaccine comparison groups	COM/COM	COM	COM	i.m/i.m	H1 and H3 IAV	10 pigs
		AUT/AUT	AUT	AUT	i.m/i.m	H1 and H3 IAV	10 pigs
		AUT/COM	AUT	COM	i.m/i.m	H1 and H3 IAV	10 pigs
		COM/AUT	COM	AUT	i.m/i.m	H1 and H3 IAV	10 pigs
		NO VAC/CHA	Saline	Saline	−/−	H1 and H3 IAV	10 pigs
	Negative control group	NO VAC/NO CHA	Saline	Saline	−/−	Saline solution	6 pigs^a^
2	Live attenuated vaccine comparison groups	LAIV/COM	LAIV	COM	i.n/i.m	H1 and H3 IAV	10 pigs
		LAIV/NONE	LAIV	None	i.n/-	H1 and H3 IAV	10 pigs

i.m: intramuscular; i.n: intranasal; IAV: influenza A virus.

^a^Three pigs from the NO VAC/NO CHA group were necropsied prior to challenge and the remaining 3 pigs were necropsied at the termination of the study (7 dpc).

### Vaccines and virus challenge preparation

All the vaccines used in this study were licensed vaccines. There were two whole inactivated vaccines (WIV) used (COM and AUT) and an LAIV. COM is a commercial quadrivalent WIV (FluSure XP^®^, Zoetis, Kalamazoo, MI) with label indication for the vaccination of healthy swine, including pregnant sows and gilts, 3 weeks of age or older as an aid in preventing respiratory disease caused by IAV subtypes H1N1, H1N2, and H3N2 Clusters IV-A and IV-B. The COM included two H3N2 strains (Cluster IV-A, Clade 3.1990.4.1 and Cluster IV-B, Clade 3.1990.4.2-3 [21]), one H1N1 strain (Clade 1A.3.2 gamma-2) and one H1N2 strain (Clade 1B.2.2.2 delta-1b). The AUT was an inactivated trivalent heterologous WIV (AgriLabs, Lincoln, NE) used as a regional autogenous vaccine by a swine veterinary clinic in the Midwest. The AUT included one H3N2 strain (Cluster human-like, Clade 3.2010.1), one H1N2 strain (Clade 1B.2.2.1 delta-1a) and one H1N1 strain (Clade 1A.3.3.3 gamma). Autogenous vaccines are, by definition, made with herd specific antigens and produced in USDA- licensed facilities. However, in comparison to commercial vaccine products, autogenous vaccine licensure requirements are somewhat less stringent as they are intended to be used in the herd-of-origin only (U.S. Code of Federal Regulations. 9 CFR 113.113 and 9 CFR 112.7). Both WIVs in this study were adjuvanted using oil-in-water adjuvants (Amphigen^®^ for COM and Emulsigen^®^-D for AUT) and the HA amino acid homology of COM and AUT vaccine is shown in Additional file 1. The LAIV was a relatively new live, attenuated, intranasal commercial IAV vaccine (Ingelvac Provenza™, Boehringer Ingelheim, St. Joseph, MO) with one H3N2 strain (Cluster I clade 3.1990.3) and one H1N1 strain (beta-gamma2-like Clade 1A.2.3-like) with an NS-1 protein truncation. All vaccines were used according to their label directions.

The two IAV challenge strains were obtained from the University of Minnesota Veterinary Diagnostic Laboratory (VDL) and were isolated from lung tissues collected from growing pigs in Minnesota which had clinical signs of respiratory disease. The H1N1 challenge strain (A/Swine/Minnesota/PAH-618/2011) had an HA belonging to the 1A.3.3.3 gamma clade. The H3N2 challenge strain (A/Swine/Minnesota/080470/2015) had an HA belonging to a human-like cluster of Clade 3.2010.1. The whole genome sequences for both challenge viruses have been deposited in GenBank with accession numbers MT377710 to MT377725. The HA protein amino acid identity of vaccine strains to challenge strains is summarized in Table 2. Virus stocks were propagated by inoculating the virus in Madin-Darby Canine Kidney (MDCK) cell-line with the Dulbecco’s modified Eagle medium (DMEM; Gibco, Life Technologies, Grand Island, NY, USA) containing the essential supplements (4% BSA fraction V 7.5% solution (Gibco, Life Technologies, Grand Island, NY, USA), 0.15% 1-mg/mL TPCK trypsin (Sigma-Aldrich, St Louis. MO. USA), and 1% antibiotic–antimycotic (Gibco, Life Technologies, Grand Island, NY, USA)) and following a common laboratory virus isolation protocol and incubated at 37 °C, 5% CO_2_ for 1 h [22]. After that, fresh medium was added and the cells were further incubated for at least 72 h until at least 80% cytopathogenic effect was observed. The media containing virus were harvested and centrifuged (1500 rpm, 10 min) and viruses were titrated on MDCK cells based on established protocols [23]. The virus titer for H1N1 challenge strain was 1 × 10^6.5 TCID_50_/mL and the titer for H3N2 challenge strain was 1 × 10^7.5 TCID_50_/mL. The harvested viruses were aliquoted and stored at − 80 °C.

**Table 2:** Hemagglutinin protein amino acid homology between vaccine and challenge strains.

Vaccine	Component (subtype)	HA clade	Amino acid identity with challenge strains (%)^a^	
			A/swine/Minnesota/PAH618/2011 (H1N1) Clade 1A3.3.3 gamma	A/Swine/Minnesota/983220-61/2016 (H3N2) Human-like 3.2010.1
COM	H1N1	Clade 1A.3.2 gamma-2	95.1	–
	H1N2	Clade 1B.2.2.2 delta-1b	78.4	–
	H3N2	Cluster IV-A 3.1990.4A	–	87.1
	H3N2	Cluster IV-B 3.1990.4B	–	88.2
AUT	H1N1	Clade 1A3.3.3 gamma	96.5	–
	H1N2	Clade 1B.2.2.1 delta-1a	78.6	–
	H3N2	Cluster human-like 3.2010.1	–	99.1
LAIV	H1N1	Clade 1A.2.3 beta-gamma2	89.2	–
	H3N2	Cluster I 3.1990.3	–	89.2

HA: hemagglutinin.

^a^ Amino acid identity of hemagglutinin proteins of vaccine and challenge strains is shown as the percentage.

### Sample collection

Blood samples were collected on arrival to the animal isolation units, 1 week after boost vaccination and at challenge. The pigs were manually restrained and 2 to 6 mL of blood was collected from the cranial vena cava using 19 to 21 gauge needles and blood collection tubes (BD Vacutainer^®^ SST™, Franklin Lakes, NJ, USA).

Nasal swabs were collected from pigs on arrival, before challenge and at days 2, 4, 5 and 6 after contact challenge with the seeder pigs using swabs (BD BBL™ Culture Swabs™, Sparks, MD, USA) inserted 2–3 cm into the pig’s nostrils and gently rotated. The nasal swabs were stored refrigerated until processing which took place within 48 h of collection.

### Necropsy and gross lung lesions

All pigs were euthanized and necropsied at 7 days post-contact (dpc) with the seeder pigs by an overdose of intravenously injected pentobarbital (FATAL-PLUS, Vortech Pharmaceuticals LTD, Dearborn, MI, USA). The percentage of gross lung lesions for each pig was analyzed and scored by a pathologist blinded to the treatments based on previously published protocols [24, 25]. Bronchoalveolar lavage fluid (BALF) was collected from each pig using a saline solution as described previously [26] (Gibco, Life Technologies, Grand Island, NY, USA).

### Determination of virus shedding

An IAV matrix gene real-time RT-PCR (RRT-PCR) was used to detect IAV shedding in all the nasal swabs and BALF samples [27]. IAV nucleic acid was extracted from the nasal swabs and BALF using the MagMAX-96 viral isolation kit (Applied Biosystems, Life Technologies, Carlsbad, CA, USA) based on the methods previously described [28]. The IAV RRT-PCR was performed using the AgPath-ID One-step RT-PCR kit (Applied Biosystems, Life Technologies, Carlsbad, CA, USA) following the established protocols [20]. A sample was considered positive by RRT-PCR if the cycle threshold (Ct) value was 38 or lower and, due to the semi-quantitative nature of the RRT-PCR test, the lower the Ct value, the higher the amount of viral RNA detected in the sample. Virus isolation was attempted on all positive nasal swabs and BALF samples on MDCK cells and titrated by calculating the TCID_50_ per mL [23]. A sample was considered positive by virus titration if the virus titer was 1 × 10^1.75 or higher.

### Library preparation and next-generation sequencing

To evaluate which strain the infected pigs were shedding, the nasal swabs and BALF samples with Ct value less than or equal to 38 (based on the matrix RT-PCR described above) were selected for next generation sequencing. One step reverse transcription-PCR amplification was performed on extracted RNA from selected samples by using SuperScript III One-Step RT-PCR system with High Fidelity Platinum Taq DNA Polymerase (Invitrogen, Life Technologies, USA) with degenerate primers (10uM MBTuni-12 M and MBTuni-13) [29]. The PCR product was visually verified by gel electrophoresis, the quality and quantity of RT-PCR product was checked by NanoDrop 1000 (Thermo Fisher Scientific). The PCR product was then cleaned up by Qiagen QIAquick PCR Purification Kit (QIAGEN, USA). The sequencing library was prepared by using the Nextera DNA XT Sample Preparation Kit (Illumina, San Diego, CA, USA) and quantified by using the Quant-iT™ PicoGreen™ dsDNA Assay Kit (Invitrogen). The barcoded libraries were pooled in equimolar concentrations and sequenced in multiplex for 150 bp paired-end on Illumina NextSeq Mid-Output Mode (130 M) at the University of Minnesota Genomics Center (UMGC). The raw contigs released from UMGC underwent quality assessment by Fast-QC [30] and then trimmed by Trimmomatic [31] to remove the adapter, barcode and low-quality sequences. The trimmed contigs were de novo assembled by Shovill [32] and the consensus sequences were annotated by FLAN [33] which is a web based influenza annotation tool available at NCBI Influenza Virus Resource.

### Hemagglutinin inhibition (HI) assay

Blood samples collected prior to challenge were tested using the hemagglutination inhibition (HI) assay according to methods previously described [34]. Before the HI assay, the serum was separated from the blood cells and the serum was treated with three parts receptor destroying enzyme (RDE; Denka Seiken, Tokyo, Japan) for 18 h at 37 °C, followed by heat inactivation at 56 °C for 30 min. After the sera cooled down to room temperature, 20% of turkey red blood cells were added and hemadsorbed for at least 30 min to remove nonspecific hemagglutinin inhibitors and natural serum agglutinins. The HI assay was performed using turkey red blood cells and the challenge strains (A/Swine/Minnesota/PAH-618/2011 H1N1 and A/Swine/Minnesota/080470/2015 H3N2) as antigens. HI antibody titer was calculated as the reciprocal of the highest serum dilution that completely inhibited hemagglutination.

### ELISPOT analysis of IFN-γ secreting cells

The ELISPOT analysis of IFN-γ secreting cells was performed on peripheral blood mononuclear cells (PBMC) collected 1 week after boost vaccination on pigs and on lymph nodes collected at necropsy. PBMCs were isolated from heparinized whole blood collected from pigs in treatment groups COM/COM, AUT/AUT, LAIV/COM, LAIV/NONE and NO VAC/NO CHA prior to challenge [35]. Lymph nodes were collected during necropsy from all vaccinated pigs. The lymph nodes were crushed to separate the cells from the tissue layers and passed through a 70-µm mesh nylon membrane (BD Falcon 352350). After treating them with ACK lysis buffer (Thermo Fisher Scientific, Grand Island, NY, USA) and washing with Hank’s balanced salt solution (HBSS; BD Falcon, Franklin Lakes, NJ, USA), the lymph node cells were ready to proceed for ELISPOT assay. An ELISPOT assay to determine IFN-γ secreting cells specific for H1 and H3 challenge strains was performed as described [36]. Briefly, 96-well ELISPOT filter plates (MAIPS4510, Millipore Corp., MA, USA) were coated overnight with porcine IFN-γ capture antibody (R&D Systems, Minneapolis, MN, USA). Freshly isolated PBMC cells (5 × 10^5 cells per well) or lymph node cells (1 × 10^6 cells per well) were loaded into duplicate wells and the stimulants (heat-inactivated H1 and H3 challenge strains) were also added into corresponding wells. After 18 h incubation (37 °C, 5% CO_2_), the porcine IFN-γ detection antibody (R&D Systems, Minneapolis, MN, USA) was added to each well and the IFN-γ secretion cells were visualized using the streptavidin-AP and NBT/BCIP for membranes (R&D systems, Minneapolis, MN, USA) and counted with an ELISPOT plate reader. Non-specific spots detected in wells coated with mock-infected RPMI (Gibco, Life Technologies, Grand Island, NY, USA) medium were subtracted from the counts of influenza-specific IFN-γ secretion cells.

### Statistical analysis

Statistical analyses were performed by comparing results of treatment groups receiving WIV (study 1) and separate analyses for comparing results from LAIV groups (study 2). The data from NO VAC/NO CHA pigs was summarized but not statistically analyzed. Quantitative RRT-PCR, virus titers from nasal swabs, weight, body temperature and influenza-specific antibody responses were analyzed by a generalized linear mixed model (GLMM) approach for repeated measures. Virus titers, HI titers, and the ELISPOT reads were log-transformed while percent lung lesions were transformed using arcsine square-roots prior to analysis. Using the R software (Version 3.4.1), transformed and non-transformed data were analyzed with a model that considered the fixed effects of treatment, day, the interaction of treatment-by-day, the random effects of room and the residual error. Day was considered the repeated factor. Treatment and treatment-by-day were assessed at the 5% level. If the treatment-by-day interaction was significant then treatment comparisons were assessed for each study day separately.

Percent lung lesions, ELISPOT counts and BALF were analyzed using a GLMM approach with a model that considered the fixed effects of treatment and the random effects of room and the residual error. Binary data were analyzed with R software (version 3.4.1) by Fisher’s Exact Test. Comparisons of LSMeans for all continuous variables were performed by the Tukey’s multiple method at the 5% level of significance. LSMeans from transformed data were back-transformed (geometric means) after analyses.

## Results

### Clinical signs and lung lesions in vaccinated pigs after challenge

**Figure 2:**
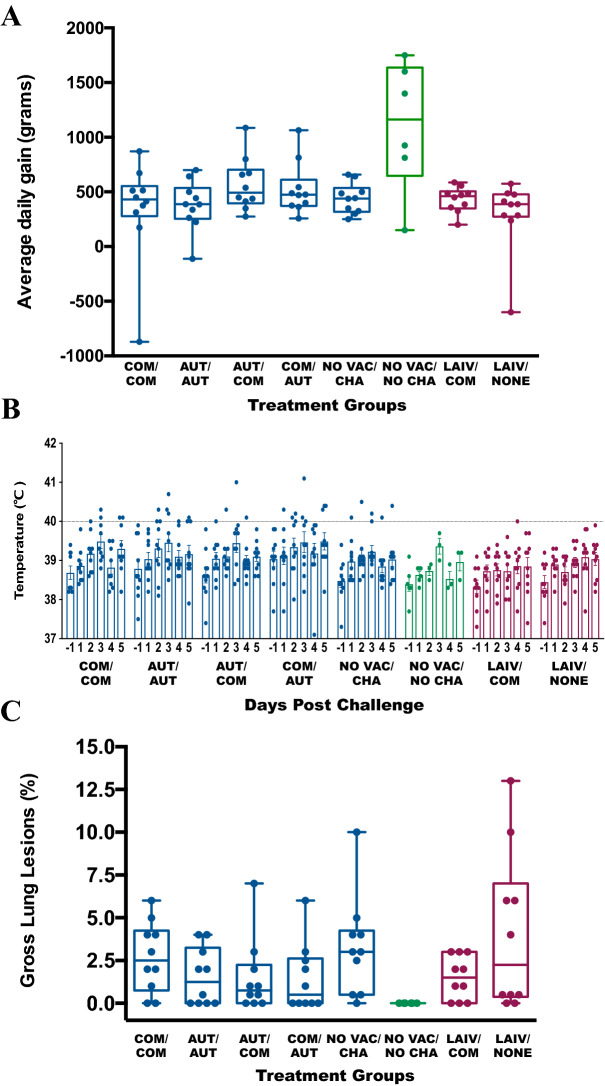
**Clinical and pathology assessment of the pigs from different treatment groups after challenge. A** Average daily weight gain (ADG) calculated from challenge to necropsy by treatment group. The ADG for pigs in multiple groups are summarized as the boxplots in grams and each data point represents an individual pig. The dark blue, green and dark red bars and data points represent the pigs from the whole inactivate vaccine comparisons, negative control and live attenuate vaccine comparison groups respectively. **B** Individual pig body temperature (°C) values by group from day prior to inoculation to necropsy. The daily body temperature (°C) for pigs in different treatment group are summarized as mean (± SEM) by bar plots and each data point represents an individual pig. The dark blue, green and dark red bars and data points represent the pigs from the whole inactivated vaccine comparisons, negative control and live attenuated vaccine comparison groups respectively. The 40.0 centigrade was set up as the threshold for fever and represented as the horizontal line. **C** The gross lung lesions for pigs at necropsy by treatment groups. The gross lung lesions for pigs are shown as percentage and presented by boxplots. Each data point indicates an individual pig. The colors for boxplots and data points are same as in **A**.

No pigs displayed any clinical signs of respiratory disease such as coughing or nasal discharge from the start of the study to necropsy. The average daily weight gain (ADG) from challenge to necropsy for the different treatment groups was measured in grams and is summarized in Figure 2A. No statistically significant differences in ADG were observed among pigs receiving the WIV combinations (*P* = 0.3062). The ADG for COM/COM pigs was 342.3 ± 147.8 g, 377.2 ± 76.7 g for AUT/AUT pigs, 567.7 ± 76.7 g for AUT/COM pigs, 524.8 ± 75.7 g for COM/AUT pigs, and 439.1 ± 43.6 g for NO VAC/CHA pigs. Also, there was no statistical difference in ADG for pigs receiving the live-attenuated vaccines. The ADG for LAIV/COM pigs was 430.9 ± 36.6 g and 293 ± 104.5 g for LAIV/NONE pigs (*P* = 0.2297). The NO VAC/NO CHA pigs had an ADG of 1106.3 ± 243.5 g.

The mean body temperatures for each treatment group shown as mean (± SEM) are summarized in Figure 2B. We considered fever present if body temperature was greater than 40 °C with no pigs having fever prior to the challenge. Three of 10 COM/COM pigs, 3/10 AUT/AUT pigs, 3/10 AUT/COM pigs, 5/10 COM/AUT pigs and 1/10 NO VAC/CHA pigs had fever after challenge. In contrast, none of the pigs receiving the LAIV vaccine (LAIV/COM and LAIV/NONE) and none of the pigs from NO VAC/NO CHA had fever during the study. There were no significant differences in body temperatures between any of the groups at any time-point (Additional file 2).

There were mild gross lung lesions in all challenged pigs in all treatment groups. The range of gross lung lesions for all vaccinated pigs ranged from 0 to 13%, the average gross lung lesions of pigs from each group were less than 5% and no significant differences were detected between treatment groups (Figure 2C).

### Virus detection in the respiratory tract

The nasal mucosa and lungs are major targets for virus replication in the upper and lower respiratory tract of pigs. Before commingling with vaccinated pigs, the seeder pigs were confirmed IAV positive by RRT-PCR on nasal swabs at day 2 post-challenge (Ct value range 21.15 to 34.19). To determine the presence and amount of virus in the respiratory tract, we collected nasal swabs at 2, 4, 5 and 6 days post-contact with the seeder pigs and BALF samples at necropsy from all pigs. The detailed infection kinetics of the individual pigs is shown in Additional file 3.

No IAV RNA was detected in any of the NO VAC/NO CHA pigs from either BALF samples or nasal swabs. Based on the BALF samples collected from pigs receiving WIV, the least amount of virus post-contact was detected in the heterologous treatment group AUT/COM (Table 3). Only a small quantity of IAV RNA was detected in the BALF sample from a single pig in this treatment group (Ct value = 34.31). The amount of viral RNA detected and the number of IAV RRT-PCR positive pigs in homologous treatment group AUT/AUT was similar to the heterologous treatment group COM/AUT. The WIV group with the highest amount of viral RNA detected was the homologous treatment group, COM/COM. Nevertheless, COM/COM pigs still had fewer positive pigs and less virus detected in BALF samples compared with the pigs from NO VAC/CHA group.

**Table 3:** Virus detection via RRT-PCR and the hemagglutinin type obtained directly from nasal swabs and bronchoalveolar lavage fluid (BALF) from pigs after contact with infected seeders and at necropsy by treatment group.

Sample type	Nasal swabs (by day)																BALF			
Treatment group	2 dpc				4 dpc				5 dpc				6 dpc				At necropsy (7 dpc)			
	PR^a^	Ct value^b^	H1^d^	H3	PR	Ct value	H1	H3	PR	Ct value	H1	H3	PR	Ct value	H1	H3	PR	Ct value	H1	H3
COM/COM	1/10	43.54 (4.61)^**c** A^	0/10	0/10	2/10	41.95 (6.67)^A^	0/10	1/10	5/10	39.06 (7.41)^A^	0/10	1/10	3/10	40.26 (8.50)^A^	0/10	1/10	6/10	32.90 (11.70)^BC^	1/10	4/10
AUT/AUT	1/10	43.66 (2.85)^A^	0/10	0/10	5/10	39.84 (5.67)^A^	1/10	0/10	5/10	38.90 (7.21)^A^	1/10	0/10	1/10	43.05 (6.16)^A^	1/10	0/10	3/10	40.74 (6.38)^A^	1/10	1/10
AUT/COM	0/10	44.39 (1.84)^A^	0/10	0/10	7/10	38.12 (5.02)^AB^	0/10	0/10	5/10	40.01 (5.41)^A^	0/10	1/10	0/10	44.35 (2.06)^A^	0/10	0/10	1/10	43.93 (3.38)^A^	0/10	0/10
COM/AUT	1/10	43.95 (3.33)^A^	0/10	0/10	5/10	39.67 (5.74)^A^	0/10	0/10	5/10	39.71 (5.76)^A^	0/10	1/10	2/10	42.18 (6.31)^A^	0/10	1/10	4/10	39.89 (6.76)^AB^	0/10	2/10
NO VAC/CHA	5/10	37.83 (8.02)^B^	1/10	3/10	6/10	33.05 (9.71)^B^	3/10	4/10	7/10	30.43 (12.09)^B^	3/10	4/10	6/10	30.72 (10.89)^B^	1/10	4/10	8/10	26.75 (10.75)^C^	7/10	4/10
NO VAC/NO CHA	0/3	45 (0)	0/3	0/3	0/3	45 (0)	0/3	0/3	0/3	45 (0)	0/3	0/3	0/3	45 (0)	0/3	0/3	0/3	45 (0)	0/3	0/3
LAIV/COM	0/10	44.25 (1.83)^A^	0/10	0/10	5/10	39.33 (6.51)^A^	0/10	1/10	5/10	39.58 (6.09)^A^	0/10	1/10	3/10	40.20 (7.76)^A^	1/10	2/10	5/10	36.29 (9.66)^A^	1/10	4/10
LAIV/NONE	7/10	36.04 (6.70)^B^	1/10	3/10	6/10	32.04 (11.36)^B^	2/10	4/10	8/10	29.56 (9.95)^B^	3/10	4/10	7/10	32.26 (8.23)^B^	2/10	4/10	10/10	23.93 (5.24)^B^	7/10	6/10

dpc: days post-contact; BALF: bronchoalveolar lavage fluid.

^a^ Positive rates (PR) are presented as the number of pigs with influenza A virus matrix gene RRT-PCR positive nasal swabs or BALF (numerator) by day post-contact (or at necropsy) out of the total number of pigs (denominator) in different treatment groups. The positive cutoff cycle threshold (Ct) value is 38.

^b^ Ct values are presented as mean (standard deviation). The actual Cts are used for samples with Ct values between 38 to 45 and the samples with Ct values above 45 are considered as 45 in the analysis.

^c^ Ct values with different superscripts (A, B, C) are significantly different (*P *< 0.05).

^d^ Number of pigs with H1 and H3 hemagglutinins detected by Next Generation Sequencing by treatment group at 2, 4, 5, and 6 days post-contact and at necropsy. Results are shown as number of positive pigs with H1 or H3 sequences/total number of pigs in the treatment group.

For the pigs receiving LAIV, all pigs in LAIV/NONE treatment group were positive by RRT-PCR and only half of the pigs in the LAIV/COM treatment group were RRT-PCR-positive, with the average Ct value for BALF samples from LAIV/NONE pigs significantly lower, e.g. more IAV viral RNA detected, than the LAIV/COM pigs (*P* < 0.0001). There was less viral RNA detectable by RRT-PCR in the nasal cavities of pigs receiving WIV combinations and, similarly, the number of pigs positive (positive rate, PR), was also lower. For the LAIV treatment groups, the LAIV/COM treatment had less infected pigs and significantly higher Ct values in both BALF samples and nasal swabs at necropsy (Table 3).

We performed next-generation sequencing on all nasal swabs and BALF samples that tested positive by RRT-PCR to determine the H1 and/or H3 gene segment in each sample (Table 3). Among the BALF samples collected from WIV pigs, no H1 or H3 genes were detected in the heterologous AUT/COM pigs. There were only H3 sequences detected in the BALF of 2 out of 10 pigs from the heterologous COM/AUT group. For the homologous WIV pigs, both HA sequences were detected in a BALF sample of 1 out of 10 AUT/AUT pigs. Based on the nasal swabs collected from WIV pigs, the HA sequences detected from heterologous AUT/COM, COM/AUT and homologous COM/COM pigs belonged to the H3 subtype. In contrast, only H1 sequences were detected in nasal swabs from homologous AUT/AUT pigs. In addition, both H1 and H3 sequences were found in nasal swabs of NO VAC/CHA pigs. Among LAIV administrated pigs, both H1 and H3 gene sequences were detected in pigs from LAIV/NONE and LAIV/COM group no matter on nasal swabs or BALF samples. Furthermore, we observed the co-infection of both challenge strains happened on multiple pigs from NO VAC/CHA and LAIV/NONE group either based on results from nasal swabs or BALF samples (Additional file 4).

### Virus titers in the respiratory tract

To better understand the shedding and transmission of the challenge viruses in the vaccinated pigs’ lungs and nasal cavities, we performed virus titration on all the BALF samples and nasal swabs collected at 2, 4 and 6 days post-contact with the seeder pigs to determine the extent of virus shedding.

**Figure 3:**
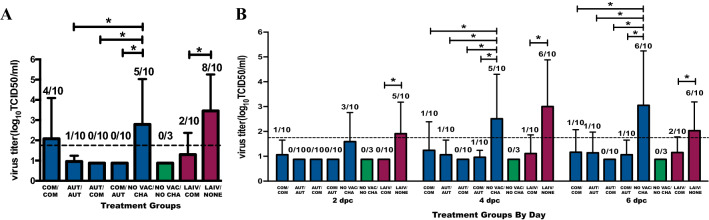
**Virus shedding in upper and lower respiratory tract from pigs by treatment group after challenge. A** Influenza A virus titer in bronchoalveolar lavage fluid (BALF) supernatant samples from vaccinated pigs at necropsy (7 days post-contact) by treatment group. **B** Influenza A virus titer of nasal swab samples collected at 2, 4 and 6 days post-contact by treatment groups. The virus titers are presented as log_10_ TCID_50_/mL and the values are shown as mean (bar) + SD (error line). The number of pigs shedding IAV is shown above each bar as a ratio of IAV positive pigs/total number of pigs tested. Pigs with virus titer above the detection limit of 1.75 TCID_50_/mL (detection limit shown as dashed line) were considered virus positive pigs. The asterisks denote the significant difference (*P* < 0.05) in virus titer between groups calculated using a generalized linear mixed model (GLM) and Tukey was used for multiple LSmeans comparisons. The dark blue, green and dark red bars and data points represent the pigs from the whole inactivate vaccine comparison, negative control and live attenuate vaccine comparison groups, respectively.

Among the WIV treatment groups, no virus was isolated from any BALF samples from heterologous prime-boost treatment pig groups COM/AUT and AUT/COM (Figure 3A). Only one pig in the homologous AUT/AUT treatment group was shedding a low quantity of virus (1.75 TCID_50_/mL). However, in the homologous COM/COM, virus was isolated in the BALF of 4/10 pigs and at higher virus concentrations than in other inactivated vaccinated groups. For the LAIV treatment groups, virus was isolated from BALF samples of 8/10 pigs in LAIV/NONE group and only 2 of the 10 pigs in the LAIV/COM group which had significantly lower average virus concentration.

The viral load in nasal swabs tested by virus isolation was in agreement with the Ct value results of the RRT-PCR. Among the WIV groups, the heterologous AUT/COM pigs had no positive pigs at any time during the study. The other three WIV treatment groups had similar nasal virus shedding kinetics (Figure 3B). Pigs in NO VAC/CHA group had significantly higher amounts of nasal virus shedding than all the WIV treatment groups on day 2 and day 4 post-contact. For the pigs from LAIV groups, the heterologous LAIV/COM treatment had significantly lower virus shedding from the nasal cavities at all selected time-points compared to that from pigs in LAIV/NONE group.

### Humoral and T-cell mediated immune responses

**Figure 4:**
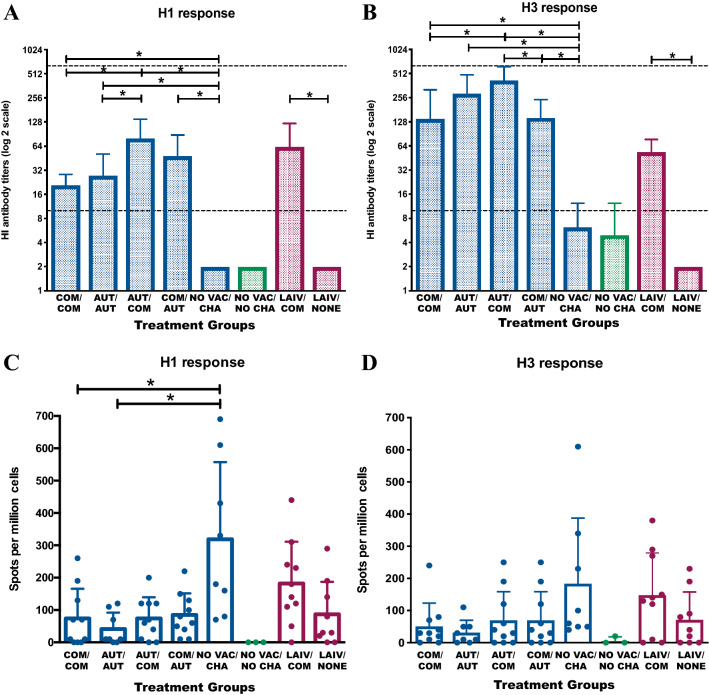
**Humoral and T-cell mediated immune response of pigs from different treatment groups.** Average HI titers in pigs against H1N1 (**A**) and H3N2 (**B**) influenza virus strains between different treatment groups before challenge. The HI titers for pigs from each treatment group are shown as mean (bar) + SD (error line). The dash lines indicate the detection limit from 1:10 to 1:640. The number of IFN-γ specific H1 (**C**) and H3 (**D**) secreting cells in pigs from lymph nodes collected at necropsy are shown by treatment group. The bars show the mean count + SD (error line) of IFN-γ secreting cell spots per million loading cells for pigs from each treatment groups. The data points indicate the spot counts for samples collected from each individual pig. Both HI titers and ELISPOT counts were log transformed first and analyzed using a generalized linear mixed model and the Tukey test was used to perform multiple LSmeans comparisons. The asterisks denote the significant difference (*P* < 0.05) of HI titers or ELISPOT counts between groups. The dark blue, green and dark red bars and/or data points represent the pigs from the whole inactivated vaccine comparisons, negative control and live attenuated vaccine comparison groups, respectively.

To quantify the antibody titers against H1 and H3 challenge strains induced by different vaccine prime-boost combinations, the HI test was performed on the sera collected from each pig prior to challenge. Consistent with the virus shedding results, the heterologous AUT/COM group had the highest antibody titers against both the H1 and H3 challenge strains (Figures 4A and B). Also, pigs in all four WIV groups had significantly higher HI antibody levels against both H1 and H3 challenge strains than pigs from the NO VAC/CHA group. Anti-H1 titers were significantly higher in heterologous AUT/COM than in the homologous prime-boost groups (e.g. COM/COM and AUT/AUT). Pigs from AUT/COM groups had significantly higher antibody levels against the H3 challenge strain than pigs from COM/COM and COM/AUT groups. The LAIV only group had no serum antibody responses detected against either H1 or H3 challenge strains. In contrast, pigs in the LAIV/COM treatment group had significantly higher antibody responses against both H1 and H3 challenge strains.

We compared the frequency of IFN-γ secreting cells between groups and assessed the antigen-specific CD8 T cell response in pigs. The cells of lymph nodes that were collected from pigs during the necropsy were stimulated with the challenge H1 or H3 viruses and the numbers of virus-specific IFN-γ secreting cells were determined by ELISPOT plate reader (Figures 4C and D). The H1 and H3 specific IFN-γ secreting cells were undetectable in NO VAC/NO CHA pigs. For the H1 response in the pigs in the WIV treatment groups, the IFN-γ secreting cell counts in NO VAC/CHA pigs were significantly higher than homologous prime-boost WIV treatment groups (COM/COM and AUT/AUT) and pigs in all four of the WIV treatment groups had similar counts of H1 IFN-γ secreting cells. The H3 IFN-γ secreting cell response was at comparable levels for all pigs in the WIV treatment groups and did not differ significantly from the NO VAC/CHA group. For pigs in the LAIV treatment groups, the COM boost vaccination preceded by LAIV prime increased the IFN-γ secreting cells against H1 and H3 challenge strains, but it did not differ significantly from pigs in the treatment group receiving a single LAIV vaccination. We also tested the frequency of virus-specific IFN-γ secreting cells in PBMC samples collected at 1 week after boost vaccination from pigs in selected groups (COM/COM, AUT/AUT, LAIV/COM and LAIV/NONE) and obtained similar results between the WIV administration groups (COM/COM and AUT/AUT). However, we observed significantly increased number of H1 and H3 specific IFN-γ secreting cells in pigs from LAIV/COM group than pigs in LAIV/NONE group (Additional file 5).

## Discussion

The advantage of heterologous prime-boost vaccination in protecting pigs and reducing influenza infections has been illustrated in a previous study [19]. To test whether this vaccination approach is applicable to commercial U.S. pig farms, we mimicked field conditions by using a seeder pig infection model where seeder pigs were infected with either an H1 or H3 virus and commingled with the vaccinated pigs to serve as challenge. The vaccinated pigs received different licensed multivalent vaccine combinations as prime and boost doses. Our results suggested that the heterologous AUT/COM prime/boost vaccine combination resulted in lower numbers of infected pigs than a WIV homologous prime/boost vaccine combination when compared to NO VAC/CHA pigs. Among the LAIV groups, lower infection levels were also observed in pigs receiving heterologous LAIV/COM prime/boost vaccination compared to a single administration of LAIV. Since the ability of vaccination to induce an immune response in pigs is an important indicator for vaccine efficacy, we also examined the humoral and cell-mediated immune responses for all vaccinated pigs receiving the different vaccine administrations. We found the heterologous prime-boost vaccination protocols may have expanded the antibody response to both H1 and H3 challenge strains as demonstrated by the higher HI titers especially in pigs from AUT/COM and LAIV/COM treatment groups. However, as expected we did not observe a significantly enhanced cell-meditated immunity in the WIV groups [37]. Overall the pigs in the treatment group that received the heterologous AUT/COM vaccination had more favorable outcomes consistent with a better response against the virus challenge than non-vaccinated pigs and compared to all the other WIV groups. The responses seen in the heterologous LAIV/COM treatment group were also more favorable than those responses observed compared to the treatment group receiving the single dose of LAIV vaccine.

Compared with traditional vaccination approaches, the goal of a heterologous prime-boost vaccination approach is two-fold in that there is not only an increase in the titer and longevity of the immune responses but also an expansion of the scope of immune responses elicited through humoral and cell-mediated immune responses [15]. As a result, heterologous prime-boost approaches have been applied against a wide range of pathogens and even complex diseases such as malaria or tuberculosis [38, 39]. The heterologous prime-boost vaccination approach was first employed in a macaque model against the simian immunodeficiency virus in the 1990s and displayed one of the most promising protection results in the early human immunodeficiency virus vaccine development effort [40]. Similar strategies to prevent influenza infections have been documented [17]. Applying heterologous prime-boost influenza regimens are thought to elicit higher levels of anti-hemagglutinin stalk antibodies which typically exhibit much broader and neutralizing activity than antibodies that bind to conventional antigenic sites on the hemagglutinin head [41]. Therefore, the heterologous prime-boost strategy may potentiate responses to suboptimal immunogens and may elicit broadly cross-reactive responses that could eliminate the need for additional vaccinations. In our study, the heterologous AUT/COM and LAIV/COM groups appeared to have the best immune responses among their comparison groups. Although we did not measure antibodies against the stalk part of the HA protein, HI titers were highest for AUT/COM in WIV groups and LAIV/COM in LAIV groups against both H1 and H3 challenge viruses, which resulted in less virus detected in pigs within these groups. We also quantified the T cell immune response for the vaccinated pigs using ELISPOT to evaluate the antigen-specific cell-mediated immune responses. Even though we did not observe a significant difference between different treatment groups, there were increased H1-specific IFN-gamma secreting cells in lymph nodes examined from pigs in the NO VAC/CHA group. This may reflect differences in IAV exposure of pigs in the different treatment groups to the seeder pigs or individual differences in the ability of the pigs to respond to non-specific challenges as exemplified by the higher counts of IFN-γ secretion cells in pigs in the NO VAC/CHA group. It is also plausible that the higher counts are due to the intense reactivity of pigs’ natural defenses against severe influenza infection [42].

The amount of virus shedding is one of the key factors used to demonstrate the extent of protection against the virus challenge afforded by vaccination. Since the IAV can replicate in both the upper and lower respiratory tract and the lung is the target organ that correlates with disease, we performed virus detection and sequencing on nasal swabs and BALF samples to evaluate not only the virus shed in nasal secretions and the amount of virus detected in the lower respiratory tract but also assessed lung pathology. Diverse infection patterns of each subtype viral population were observed in pigs from homologous and heterologous WIV groups, and in multiple pigs from NO VAC/CHA and LAIV/NONE groups shedding both challenge viruses through the lungs and nasal cavities. In our study, we used a seeder pig model that intended to mimic transmission via nose-to-nose contact instead of direct intranasal or intratracheal inoculation of pigs with high quantities of challenge virus as these direct inoculation methods are not an accurate reflection of what occurs naturally [43, 44]. Furthermore, direct intratracheal challenge is a consistent factor in vaccination/challenge studies wherein enhanced respiratory tract pathology is observed [45, 46]. Meanwhile, high dose intranasal challenge could cause high virus nasal shedding at the next day after challenge which represents a problem for vaccine evaluation, especially when evaluating T cell-inducing vaccines which usually have poor ability to prevent the virus entry into cells [44].

Pigs from the LAIV vaccine treatment groups were not housed in the same rooms as pigs in the WIV treatment groups because the LAIV vaccine is shed for a short period of time after administration (https://www.ingelvacprovenza.com/additional-data). As a result, direct comparisons between pigs in the WIV treatment groups to pigs in the LAIV treatment groups could not be made. All pigs receiving LAIV vaccine were confirmed IAV RRT-PCR negative on nasal swabs collected prior to challenge and the hemagglutination sequences obtained from the IAV positive samples all belonged to the challenge viruses. Thus, we can conclude that the virus shed after challenge is the challenge strain rather than the vaccine strain. We found the heterologous LAIV/COM pigs shed reduced levels of virus in both the upper and lower respiratory tract compared with LAIV/NONE pigs. Among the WIV groups, the heterologous AUT/COM vaccination may be said to offer the best protection since no virus was isolated from any BALF samples or nasal swabs from pigs in this group. Even though we did not detect significant differences in virus shedding with the COM/AUT group, this group had lower HI titers compared to the AUT/COM pigs. Although the limited number of pigs in each treatment group may have reduced the sensitivity of our study, thereby impairing our ability to detect differences between vaccination protocols, there remain questions regarding the order of the vaccine used in heterologous prime-boost vaccinations and whether the benefits imparted by vaccination are dependent on which vaccine is used as the prime and which vaccine is used as the boost. Which vaccine is first and which is second may be irrelevant. If, perhaps, order does affect heterologous prime-boost vaccination performance, then this order “phenomenon” seen in our study and also present in other influenza vaccination studies [19] may be explained by the assumption that the heterologous prime-boost favors the antibody response against the first encountered antigen(s) without impairing the immune response to the second encountered antigen(s), since in our study, the HA protein of the AUT vaccine strains shared the highest amino acid identity with the HA of the challenge viruses. Moreover, this is also supported by the concept of “back-boosting” which refers to the tendency of the immune system during second influenza exposures to boost the titers of antibodies against the previous encountered vaccine strains [47, 48]. The variability of HA homology between the different vaccines and the challenge strains could also lead to differences on results between homologous WIV groups. However, it was not the main aim of the study to compare the differences between the homologous vaccinated groups but rather to compare as a whole the differences between homologous and heterologous vaccinated groups. Nevertheless, the key question remains whether this assumption applies to any heterologous prime-boost scenario; thus, the order and delivery of multivalent vaccine combinations is still an area that needs further investigation.

The seeder pig model, while closely mimicking on-farm exposure to influenza, is often challenging to perform. One difficulty was the variability encountered in virus shedding from seeder pigs for a duration of sufficient length to uniformly expose in-contact pigs. However, the effects on the overall study results due to this were mitigated given that each room contained animals from all WIV vaccination treatment groups, thereby minimizing the impact of the seeder and/or room effect. The distribution of pigs from all treatments in each of the rooms was a strength of the study and, though increasing the study complexity, was helpful in minimizing the seeder and/or room effect.

Control of IAV in pigs is complicated, and thorough evaluation of available vaccines for pigs in a controlled vaccine/challenge experiment that mimics field conditions is necessary. To accomplish this, we chose virus challenge strains that represent the IAV subtypes and clades most commonly detected in U.S. swine IAV surveillance [49], used a seeder-pig model to simultaneously exposed pigs to both IAV challenge strains, and vaccinated pigs with vaccines that are commercially licensed and ready-to-use in U.S. pig populations. In summary, we demonstrated the advantage of applying a heterologous vaccine combination using inactivated vaccines against simultaneous exposure to two IAV subtypes. Meanwhile, delivering a whole inactivated vaccine boost to pigs following a single priming administration of LAIV also significantly improved protection that may be imparted by vaccines, as was evident in decreased virus detected and increased immune response. The potential to apply this vaccination strategy to pigs in the United States is appealing. More studies are still needed to validate the concept of heterologous prime-boost to control IAV under field conditions.

## Supplementary information

**Additional file 1. Hemagglutinin protein amino acid homology between COM and AUT vaccine strains.**

**Additional file 2. Summary of body temperatures of pigs by treatment groups.**

**Additional file 3. Virus detection via RRT-PCR on nasal swabs and BALF samples from each individual pig before and after contact.**

**Additional file 4. Hemagglutinin sequences detected by next generation sequencing from nasal swabs and BALF from individual pigs after contact with infected seeder pigs.**

**Additional file 5. The number of IFN-γ specific H1 (A) and H3 (B) secreting cells in pigs from peripheral blood mononuclear cells (PBMC) collected at necropsy by treatment group.** The number of IFN-γ secreting cells in each treatment group are summarized as mean ± SD. The spot counts for individual pigs are displayed as data points. The asterisks denote the significant difference (*P* < 0.05) of the number of IFN-γ secreting cell spots between groups. The dark blue, green and dark red bars and/or data points represent the pigs from the whole inactivate vaccine comparisons, negative control and live attenuate vaccine comparisons, respectively.

## Data Availability

All data generated or analyzed during this study are included in this published article.

## Abbreviations

IAV: influenza A virus
US: United States
LAIV: live attenuated IAV vaccine
PRRSV: porcine reproductive and respiratory syndrome virus
*M. hyopneumoniae*: *Mycoplasma hyopneumoniae*
ADCC: antibody-dependent cellular cytotoxicity
WIV: whole-cell inactivated vaccines
COM: commercial vaccine
AUT: autogenous vaccine
NO VAC/CHA: no vaccination but challenged
NONE: no boost vaccination
RRT-PCR: real-time reverse transcriptase-polymerase chain reaction
VDL: veterinary Diagnostic Laboratory
MDCK: Madin-Darby Canine Kidney cell-line
DMEM: Dulbecco’s modified Eagle medium
BALF: bronchoalveolar lavage fluid
Ct: cycle threshold
HI: hemagglutination inhibition assay
RDE: receptor destroying enzyme
PBMC: peripheral blood mononuclear cells
ADG: average daily weight gain
i.m: intramuscular
i.n: intranasal
