# Supplementary material for: Exploring heterologous prime-boost vaccination approaches to enhance influenza control in pigs
Source: Vet Res. 2020 Jul 9;51:89. doi: 10.1186/s13567-020-00810-z (PMC7344353; doi:10.1186/s13567-020-00810-z)
Supplement: Supplementary file 1 — Additional file 1. Hemagglutinin protein amino acid homology between COM and AUT vaccine strains. [file 13567_2020_810_MOESM1_ESM.docx]

| **Vaccine** | **Component (subtype)** | **HA^a^ clade** | AUT (%)^b^ | | |
| --- | --- | --- | --- | --- | --- |
|  |  |  | H1N1 | H1N2 | H3N2 |
|  |  |  | Clade 1A3.3.3 gamma | Clade 1B.2.2.1 delta-1a | Cluster human-like 3.2010.1 |
| COM | H1N1 | Clade 1A.3.2 gamma - 2 | 93.1 | 78.3 | - |
|  | H1N2 | Clade 1B.2.2.2 delta - 1b | 78.4 | 94.0 | - |
|  | H3N2 | Cluster IV-A 3.1990.4A | - | - | 86.9 |
|  | H3N2 | Cluster IV-B 3.1990.4B | - | - | 88.0 |

Table S1. Hemagglutinin protein amino acid homology between COM and AUT vaccine strains.

^a.^ HA: hemagglutinin.

^b.^ Amino acid identity of hemagglutinin proteins of vaccine and challenge strains is shown as the percentage.
