# Supplementary material for: Exploring heterologous prime-boost vaccination approaches to enhance influenza control in pigs
Source: Vet Res. 2020 Jul 9;51:89. doi: 10.1186/s13567-020-00810-z (PMC7344353; doi:10.1186/s13567-020-00810-z)
Supplement: Supplementary file 2 — Additional file 2. Summary of body temperatures of pigs by treatment groups. [file 13567_2020_810_MOESM2_ESM.docx]

Table S2. Summary of body temperatures of pigs by treatment groups.

| **Treatment group** | **N ^a^** | **Body temperature (℃) ^b^**  **Day post-contact (dpc)** | | | | | |
| --- | --- | --- | --- | --- | --- | --- | --- |
|  |  | **0 dpc** | **2 dpc** | **3 dpc** | **4 dpc** | **5 dpc** | **6 dpc** |
| COM/COM | 10 | 38.69 (0.49) | 38.87 (0.44) | 39.18 (0.49) | 39.49 (0.53) | 38.82 (0.51) | 39.30 (0.64) |
| AUT/AUT | 10 | 38.79 (0.74) | 39.04 (0.53) | 39.31 (0.74) | 39.46 (0.74) | 39.12 (0.49) | 39.17 (0.69) |
| AUT/COM | 10 | 38.63 (0.61) | 39.05 (0.50) | 39.11 (0.47) | 39.45 (0.76) | 38.98 (0.46) | 39.10 (0.39) |
| COM/AUT | 10 | 39.04 (0.60) | 39.12 (0.71) | 39.35 (0.71) | 39.47 (0.84) | 39.18 (0.83) | 39.38 (0.78) |
| NO VAC/CHA | 10 | 38.48 (0.51) | 38.98 (0.52) | 39.09 (0.55) | 39.23 (0.49) | 38.85 (0.51) | 39.03 (0.57) |
| NO VAC/NO CHA | 3 | 38.67 (0.33) | 38.80 (0.00) | 38.73 (0.17) | 39.37 (0.29) | 38.90 (0.41) | 38.97 (0.33) |
| LAIV/NONE | 10 | 38.45 (0.49) | 38.90 (0.33) | 38.71 (0.40) | 38.95 (0.41) | 39.09 (0.61) | 39.04 (0.53) |
| LAIV/COM | 10 | 38.33 (0.51) | 38.73 (0.50) | 38.76 (0.49) | 38.73 (0.54) | 38.86 (0.63) | 38.85 (0.72) |

^a^. The N present the total number of pigs from each treatment group that include in the statistical analysis.

^b^. The values of temperature are presented as mean (standard deviation).
