# Supplementary material for: Exploring heterologous prime-boost vaccination approaches to enhance influenza control in pigs
Source: Vet Res. 2020 Jul 9;51:89. doi: 10.1186/s13567-020-00810-z (PMC7344353; doi:10.1186/s13567-020-00810-z)
Supplement: Supplementary file 3 — Additional file 3. Virus detection via RRT-PCR on nasal swabs and BALF samples from each individual pig before and after contact. [file 13567_2020_810_MOESM3_ESM.docx]

Table S3. Virus detection via RRT-PCR on nasal swabs and BALF samples from each individual pig before and after contact.

| **Pig ID** | **Treatment group** | **Nasal swab (by day)**  **Ct value** | | | | | | **BALF**  **Ct value** |
| --- | --- | --- | --- | --- | --- | --- | --- | --- |
|  |  | **-1 dpc^a^** | **0 dpc** | **2 dpc** | **4 dpc** | **5 dpc** | **6 dpc** | **7 dpc** |
| 4494 | NO VAC/NO CHA | Neg^b^ | Neg | Neg | Neg | Neg | Neg | Neg |
| 5171 | NO VAC/NO CHA | Neg | Neg | Neg | Neg | Neg | Neg | Neg |
| 5193 | NO VAC/NO CHA | Neg | Neg | Neg | Neg | Neg | Neg | Neg |
| 4476 | NO VAC/NO CHA | Neg | NA | NA | NA | NA | NA | NA |
| 4935 | NO VAC/NO CHA | Neg | NA | NA | NA | NA | NA | NA |
| 5180 | NO VAC/NO CHA | Neg | NA | NA | NA | NA | NA | NA |
| 4499 | COM/COM | Neg | Neg | Neg | Neg | 35.23 | 33.02 | 27.54 |
| 5184 | COM/COM | Neg | Neg | Neg | Neg | Neg | 34.48 | 17.83 |
| 4939 | COM/COM | Neg | Neg | Neg | Neg | Neg | Neg | Neg |
| 5194 | COM/COM | Neg | Neg | Neg | Neg | Neg | Neg | Neg |
| 4482 | COM/COM | Neg | Neg | Neg | Neg | 36.11 | Neg | 36.67 |
| 5175 | COM/COM | Neg | Neg | Neg | Neg | Neg | Neg | 27.58 |
| 4471 | COM/COM | Neg | Neg | 30.42 | 33.45 | 34.59 | Neg | 19.51 |
| 4551 | COM/COM | Neg | Neg | Neg | 26.03 | 22.61 | 20.1 | 19.89 |
| 4491 | COM/COM | Neg | Neg | Neg | Neg | Neg | Neg | Neg |
| 4948 | COM/COM | Neg | Neg | Neg | Neg | 37.08 | Neg | Neg |
| 4474 | AUT/AUT | Neg | Neg | Neg | 36.34 | Neg | Neg | 37.50 |
| 4488 | AUT/AUT | Neg | Neg | Neg | 35.17 | 35.97 | Neg | Neg |
| 4478 | AUT/AUT | Neg | Neg | Neg | Neg | Neg | Neg | Neg |
| 5173 | AUT/AUT | Neg | Neg | Neg | Neg | Neg | Neg | Neg |
| 4472 | AUT/AUT | Neg | Neg | Neg | Neg | 37.05 | Neg | Neg |
| 4496 | AUT/AUT | Neg | Neg | Neg | Neg | Neg | Neg | Neg |
| 4486 | AUT/AUT | Neg | Neg | Neg | 35.74 | 32.15 | Neg | 26.12 |
| 5183 | AUT/AUT | Neg | Neg | 37.35 | 35.77 | 34.08 | Neg | Neg |
| 4477 | AUT/AUT | Neg | Neg | Neg | 30.42 | 24.73 | 25.53 | 35.62 |
| 4489 | AUT/AUT | Neg | Neg | Neg | Neg | Neg | Neg | Neg |
| 4941 | AUT/COM | Neg | Neg | Neg | 34.09 | 35.75 | Neg | Neg |
| 5170 | AUT/COM | Neg | Neg | Neg | 35.21 | 35.00 | Neg | 34.31 |
| 4932 | AUT/COM | Neg | Neg | Neg | Neg | Neg | Neg | Neg |
| 5186 | AUT/COM | Neg | Neg | Neg | 36.83 | Neg | Neg | Neg |
| 4466 | AUT/COM | Neg | Neg | Neg | 36.51 | 37.79 | Neg | Neg |
| 4940 | AUT/COM | Neg | Neg | Neg | 37.41 | Neg | Neg | Neg |
| 4468 | AUT/COM | Neg | Neg | Neg | 31.60 | 34.05 | Neg | Neg |
| 5181 | AUT/COM | Neg | Neg | Neg | 34.55 | 32.56 | Neg | Neg |
| 5188 | AUT/COM | Neg | Neg | Neg | Neg | Neg | Neg | Neg |
| 5189 | AUT/COM | Neg | Neg | Neg | Neg | Neg | Neg | Neg |
| 4465 | COM/AUT | Neg | Neg | Neg | 34.35 | Neg | Neg | 30.66 |
| 4550 | COM/AUT | Neg | Neg | Neg | 36.35 | 35.36 | Neg | Neg |
| 4554 | COM/AUT | Neg | Neg | Neg | Neg | Neg | Neg | Neg |
| 4930 | COM/AUT | Neg | Neg | Neg | Neg | Neg | Neg | Neg |
| 4942 | COM/AUT | Neg | Neg | Neg | Neg | 36.97 | Neg | Neg |
| 4946 | COM/AUT | Neg | Neg | Neg | 35.80 | Neg | Neg | Neg |
| 4467 | COM/AUT | Neg | Neg | 34.48 | 33.00 | 31.29 | 35.37 | 30.88 |
| 4931 | COM/AUT | Neg | Neg | Neg | 32.18 | 33.33 | 26.43 | 31.31 |
| 5177 | COM/AUT | Neg | Neg | Neg | Neg | Neg | Neg | 36.05 |
| 5178 | COM/AUT | Neg | Neg | Neg | Neg | 35.17 | Neg | Neg |
| 4473 | LAIV/NONE | Neg | Neg | 31.68 | 27.59 | 22.46 | 24.50 | 21.46 |
| 4475 | LAIV/NONE | Neg | Neg | Neg | Neg | 34.22 | Neg | 33.75 |
| 4492 | LAIV/NONE | Neg | Neg | Neg | Neg | Neg | Neg | 29.84 |
| 4934 | LAIV/NONE | Neg | Neg | Neg | Neg | Neg | Neg | 28.25 |
| 4937 | LAIV/NONE | Neg | Neg | 37.52 | Neg | 37.09 | 35.86 | 22.62 |
| 4479 | LAIV/NONE | Neg | Neg | 28.35 | 24.70 | 19.46 | 27.15 | 24.28 |
| 4481 | LAIV/NONE | Neg | Neg | 35.35 | 24.21 | 25.77 | 25.33 | 23.45 |
| 4484 | LAIV/NONE | Neg | Neg | 31.49 | 23.50 | 20.90 | 26.45 | 17.76 |
| 4945 | LAIV/NONE | Neg | Neg | 30.87 | 19.40 | 21.41 | 27.65 | 19.77 |
| 5166 | LAIV/NONE | Neg | Neg | 30.10 | 20.96 | 24.34 | 26.43 | 18.15 |
| 4483 | LAIV/COM | Neg | Neg | Neg | Neg | 37.22 | 27.71 | Neg |
| 4498 | LAIV/COM | Neg | Neg | Neg | Neg | Neg | Neg | Neg |
| 4949 | LAIV/COM | Neg | Neg | Neg | Neg | Neg | Neg | Neg |
| 5172 | LAIV/COM | Neg | Neg | Neg | Neg | Neg | Neg | Neg |
| 5191 | LAIV/COM | Neg | Neg | Neg | Neg | Neg | Neg | Neg |
| 4469 | LAIV/COM | Neg | Neg | Neg | 35.24 | 36.91 | Neg | 26.18 |
| 4552 | LAIV/COM | Neg | Neg | Neg | 34.75 | 34.69 | Neg | 29.85 |
| 4933 | LAIV/COM | Neg | Neg | Neg | 27.29 | 30.48 | 28.51 | 20.30 |
| 5182 | LAIV/COM | Neg | Neg | Neg | 37.52 | Neg | Neg | 30.94 |
| 5185 | LAIV/COM | Neg | Neg | Neg | 33.47 | 31.24 | 30.79 | 30.63 |
| 5174 | NO VAC/CHA | Neg | Neg | 29.02 | 25.34 | 16.39 | 18.06 | 16.60 |
| 5179 | NO VAC/CHA | Neg | Neg | 29.07 | 22.97 | 24.99 | 19.96 | 17.58 |
| 4553 | NO VAC/CHA | Neg | Neg | Neg | Neg | Neg | Neg | Neg |
| 5192 | NO VAC/CHA | Neg | Neg | Neg | Neg | Neg | Neg | Neg |
| 4495 | NO VAC/CHA | Neg | Neg | 31.18 | 25.28 | 25.52 | 30.90 | 24.46 |
| 4944 | NO VAC/CHA | Neg | Neg | Neg | Neg | 37.37 | Neg | 29.32 |
| 4490 | NO VAC/CHA | Neg | Neg | 37.24 | 22.36 | 15.30 | 19.50 | 14.97 |
| 5167 | NO VAC/CHA | Neg | Neg | 26.75 | 25.79 | 18.18 | 19.26 | 20.89 |
| 4493 | NO VAC/CHA | Neg | Neg | Neg | 35.73 | 31.58 | 32.35 | 25.98 |
| 4938 | NO VAC/CHA | Neg | Neg | Neg | Neg | Neg | Neg | 27.73 |
| 4470 | H1 Seeder | Neg | 31.06 | 27.59 | Neg | Neg | Neg | Neg |
| 4485 | H1 Seeder | Neg | 27.51 | 35.33 | 37.58 | 37.33 | Neg | Neg |
| 4487 | H1 Seeder | Neg | 30.51 | 32.43 | 35.76 | Neg | 37.96 | 34.37 |
| 4497 | H1 Seeder | 34.49 | 27.10 | 20.65 | 26.25 | 27.21 | 23.21 | 19.26 |
| 5168 | H1 Seeder | 30.70 | 24.56 | 24.29 | 25.60 | 29.44 | 34.62 | 23.26 |
| 5169 | H1 Seeder | Neg | 33.56 | Neg | 35.10 | 30.72 | Neg | Neg |
| 5190 | H1 Seeder | 29.44 | 25.10 | 30.27 | 30.87 | Neg | Neg | Neg |
| 4936 | H3 Seeder | Neg | 34.19 | Neg | Neg | 37.62 | Neg | 31.04 |
| 4947 | H3 Seeder | 26.21 | 23.16 | 30.39 | 31.00 | 31.89 | 36.49 | 31.17 |
| 4480 | H3 Seeder | 25.29 | 21.15 | 26.21 | 29.18 | 32.21 | 28.85 | 18.14 |
| 5176 | H3 Seeder | 27.18 | 25.14 | 25.1 | 30.80 | 34.14 | 35.1 | 30.86 |
| 4943 | H3 Seeder | Neg | 33.35 | Neg | Neg | Neg | Neg | Neg |
| 5187 | H3 Seeder | 31.67 | 27.54 | 33.08 | 35.67 | 36.75 | Neg | Neg |
| 5165 | H3 Seeder | 36.39 | 29.65 | Neg | Neg | Neg | Neg | Neg |

^a^ Abbreviations: dpc: days post-contact (dpc is in reference of when challenged seeder pigs were placed in contact with the rest of the pigs); Neg: negative; NC: not applicable.

^b^ Samples with Ct value above 38 are considered as negative.
