# Supplementary material for: Exploring heterologous prime-boost vaccination approaches to enhance influenza control in pigs
Source: Vet Res. 2020 Jul 9;51:89. doi: 10.1186/s13567-020-00810-z (PMC7344353; doi:10.1186/s13567-020-00810-z)
Supplement: Supplementary file 4 — Additional file 4. Hemagglutinin sequences detected by next generation sequencing from nasal swabs and BALF from individual pigs after contact with infected seeder pigs. [file 13567_2020_810_MOESM4_ESM.docx]

Table S4. Hemagglutinin sequences detected by next generation sequencing from nasal swabs and BALF from individual pigs after contact with infected seeder pigs.

| **Pig ID** | **Treatment Group** | **Nasal swab (by day)** | | | | | | | | **BALF** | |
| --- | --- | --- | --- | --- | --- | --- | --- | --- | --- | --- | --- |
|  |  | **2 dpc^a^** | | **4 dpc** | | **5 dpc** | | **6 dpc** | | **7 dpc** | |
|  |  | **H1** | **H3** | **H1** | **H3** | **H1** | **H3** | **H1** | **H3** | **H1** | **H3** |
| 4494 | NO VAC/NO CHA | **NA^ac^** | **NA** | **NA** | **NA** | **NA** | **NA** | **NA** | **NA** | **NA** | **NA** |
| 5171 | NO VAC/NO CHA | **NA** | **NA** | **NA** | **NA** | **NA** | **NA** | **NA** | **NA** | **NA** | **NA** |
| 5193 | NO VAC/NO CHA | **NA** | **NA** | **NA** | **NA** | **NA** | **NA** | **NA** | **NA** | **NA** | **NA** |
| 4476 | NO VAC/NO CHA | **NA** | **NA** | **NA** | **NA** | **NA** | **NA** | **NA** | **NA** | **NA** | **NA** |
| 4935 | NO VAC/NO CHA | **NA** | **NA** | **NA** | **NA** | **NA** | **NA** | **NA** | **NA** | **NA** | **NA** |
| 5180 | NO VAC/NO CHA | **NA** | **NA** | **NA** | **NA** | **NA** | **NA** | **NA** | **NA** | **NA** | **NA** |
| 4499 | COM/COM | **NA** | **NA** | **NA** | **NA** | **Neg^a^** | **Neg** | **Neg** | **Neg** | **Neg** | **Pos^ab^** |
| 5184 | COM/COM | **NA** | **NA** | **NA** | **NA** | **NA** | **NA** | **Neg** | **Neg** | **Neg** | **Pos** |
| 4939 | COM/COM | **NA** | **NA** | **NA** | **NA** | **NA** | **NA** | **NA** | **NA** | **NA** | **NA** |
| 5194 | COM/COM | **NA** | **NA** | **NA** | **NA** | **NA** | **NA** | **NA** | **NA** | **NA** | **NA** |
| 4482 | COM/COM | **NA** | **NA** | **NA** | **NA** | **Neg** | **Neg** | **NA** | **NA** | **Neg** | **Neg** |
| 5175 | COM/COM | **NA** | **NA** | **NA** | **NA** | **NA** | **NA** | **NA** | **NA** | **Pos** | **Neg** |
| 4471 | COM/COM | **Neg** | **Neg** | **Neg** | **Neg** | **Neg** | **Neg** | **NA** | **NA** | **Neg** | **Pos** |
| 4551 | COM/COM | **NA** | **NA** | **Neg** | **Pos** | **Neg** | **Pos** | **Neg** | **Pos** | **Neg** | **Pos** |
| 4491 | COM/COM | **NA** | **NA** | **NA** | **NA** | **NA** | **NA** | **NA** | **NA** | **NA** | **NA** |
| 4948 | COM/COM | **NA** | **NA** | **NA** | **NA** | **Neg** | **Neg** | **NA** | **NA** | **NA** | **NA** |
| 4474 | AUT/AUT | **NA** | **NA** | **Neg** | **Neg** | **NA** | **NA** | **NA** | **NA** | **Neg** | **Neg** |
| 4488 | AUT/AUT | **NA** | **NA** | **Neg** | **Neg** | **Neg** | **Neg** | **NA** | **NA** | **NA** | **NA** |
| 4478 | AUT/AUT | **NA** | **NA** | **NA** | **NA** | **NA** | **NA** | **NA** | **NA** | **NA** | **NA** |
| 5173 | AUT/AUT | **NA** | **NA** | **NA** | **NA** | **NA** | **NA** | **NA** | **NA** | **NA** | **NA** |
| 4472 | AUT/AUT | **NA** | **NA** | **NA** | **NA** | **Neg** | **Neg** | **NA** | **NA** | **NA** | **NA** |
| 4496 | AUT/AUT | **NA** | **NA** | **NA** | **NA** | **NA** | **NA** | **NA** | **NA** | **NA** | **NA** |
| 4486 | AUT/AUT | **NA** | **NA** | **Neg** | **Neg** | **Neg** | **Neg** | **NA** | **NA** | **Pos** | **Pos** |
| 5183 | AUT/AUT | **Neg** | **Neg** | **Neg** | **Neg** | **Neg** | **Neg** | **NA** | **NA** | **NA** | **NA** |
| 4477 | AUT/AUT | **NA** | **NA** | **Pos** | **Neg** | **Pos** | **Neg** | **Pos** | **Neg** | **Neg** | **Neg** |
| 4489 | AUT/AUT | **NA** | **NA** | **NA** | **NA** | **NA** | **NA** | **NA** | **NA** | **NA** | **NA** |
| 4941 | AUT/COM | **NA** | **NA** | **Neg** | **Neg** | **Neg** | **Neg** | **NA** | **NA** | **NA** | **NA** |
| 5170 | AUT/COM | **NA** | **NA** | **Neg** | **Neg** | **Neg** | **Neg** | **NA** | **NA** | **Neg** | **Neg** |
| 4932 | AUT/COM | **NA** | **NA** | **NA** | **NA** | **NA** | **NA** | **NA** | **NA** | **NA** | **NA** |
| 5186 | AUT/COM | **NA** | **NA** | **Neg** | **Neg** | **NA** | **NA** | **NA** | **NA** | **NA** | **NA** |
| 4466 | AUT/COM | **NA** | **NA** | **Neg** | **Neg** | **Neg** | **Neg** | **NA** | **NA** | **NA** | **NA** |
| 4940 | AUT/COM | **NA** | **NA** | **Neg** | **Neg** | **NA** | **NA** | **NA** | **NA** | **NA** | **NA** |
| 4468 | AUT/COM | **NA** | **NA** | **Neg** | **Neg** | **Neg** | **Neg** | **NA** | **NA** | **NA** | **NA** |
| 5181 | AUT/COM | **NA** | **NA** | **Neg** | **Neg** | **Neg** | **Pos** | **NA** | **NA** | **NA** | **NA** |
| 5188 | AUT/COM | **NA** | **NA** | **NA** | **NA** | **NA** | **NA** | **NA** | **NA** | **NA** | **NA** |
| 5189 | AUT/COM | **NA** | **NA** | **NA** | **NA** | **NA** | **NA** | **NA** | **NA** | **NA** | **NA** |
| 4465 | COM/AUT | **NA** | **NA** | **Neg** | **Neg** | **NA** | **NA** | **NA** | **NA** | **Neg** | **Neg** |
| 4550 | COM/AUT | **NA** | **NA** | **Neg** | **Neg** | **Neg** | **Neg** | **NA** | **NA** | **NA** | **NA** |
| 4554 | COM/AUT | **NA** | **NA** | **NA** | **NA** | **NA** | **NA** | **NA** | **NA** | **NA** | **NA** |
| 4930 | COM/AUT | **NA** | **NA** | **NA** | **NA** | **NA** | **NA** | **NA** | **NA** | **NA** | **NA** |
| 4942 | COM/AUT | **NA** | **NA** | **NA** | **NA** | **Neg** | **Neg** | **NA** | **NA** | **NA** | **NA** |
| 4946 | COM/AUT | **NA** | **NA** | **Neg** | **Neg** | **NA** | **NA** | **NA** | **NA** | **NA** | **NA** |
| 4467 | COM/AUT | **Neg** | **Neg** | **Neg** | **Neg** | **Neg** | **Neg** | **Neg** | **Neg** | **Neg** | **Pos** |
| 4931 | COM/AUT | **NA** | **NA** | **Neg** | **Neg** | **Neg** | **Pos** | **Neg** | **Pos** | **Neg** | **Pos** |
| 5177 | COM/AUT | **NA** | **NA** | **NA** | **NA** | **NA** | **NA** | **NA** | **NA** | **Neg** | **Neg** |
| 5178 | COM/AUT | **NA** | **NA** | **NA** | **NA** | **Neg** | **Neg** | **NA** | **NA** | **NA** | **NA** |
| 4473 | LAIV/NONE | **Pos** | **Neg** | **Pos** | **Neg** | **Pos** | **Neg** | **Pos** | **Neg** | **Pos** | **Neg** |
| 4475 | LAIV/NONE | **NA** | **NA** | **NA** | **NA** | **Neg** | **Neg** | **NA** | **NA** | **Neg** | **Neg** |
| 4492 | LAIV/NONE | **NA** | **NA** | **NA** | **NA** | **NA** | **NA** | **NA** | **NA** | **Pos** | **Neg** |
| 4934 | LAIV/NONE | **NA** | **NA** | **NA** | **NA** | **NA** | **NA** | **NA** | **NA** | **Pos** | **Pos** |
| 4937 | LAIV/NONE | **Neg** | **Neg** | **NA** | **NA** | **Neg** | **Neg** | **Neg** | **Neg** | **Pos** | **Neg** |
| 4479 | LAIV/NONE | **Neg** | **Pos** | **Neg** | **Pos** | **Neg** | **Pos** | **Neg** | **Pos** | **Pos** | **Pos** |
| 4481 | LAIV/NONE | **Neg** | **Neg** | **Pos** | **Neg** | **Pos** | **Neg** | **Pos** | **Neg** | **Pos** | **Pos** |
| 4484 | LAIV/NONE | **Neg** | **Pos** | **Neg** | **Pos** | **Pos** | **Pos** | **Neg** | **Pos** | **Neg** | **Pos** |
| 4945 | LAIV/NONE | **Neg** | **Neg** | **Neg** | **Pos** | **Neg** | **Pos** | **Neg** | **Pos** | **Neg** | **Pos** |
| 5166 | LAIV/NONE | **Neg** | **Pos** | **Neg** | **Pos** | **Neg** | **Pos** | **Neg** | **Pos** | **Pos** | **Pos** |
| 4483 | LAIV/COM | **NA** | **NA** | **NA** | **NA** | **Neg** | **Neg** | **Pos** | **Neg** | **NA** | **NA** |
| 4498 | LAIV/COM | **NA** | **NA** | **NA** | **NA** | **NA** | **NA** | **NA** | **NA** | **NA** | **NA** |
| 4949 | LAIV/COM | **NA** | **NA** | **NA** | **NA** | **NA** | **NA** | **NA** | **NA** | **NA** | **NA** |
| 5172 | LAIV/COM | **NA** | **NA** | **NA** | **NA** | **NA** | **NA** | **NA** | **NA** | **NA** | **NA** |
| 5191 | LAIV/COM | **NA** | **NA** | **NA** | **NA** | **NA** | **NA** | **NA** | **NA** | **NA** | **NA** |
| 4469 | LAIV/COM | **NA** | **NA** | **Neg** | **Neg** | **Neg** | **Neg** | **NA** | **NA** | **Neg** | **Pos** |
| 4552 | LAIV/COM | **NA** | **NA** | **Neg** | **Neg** | **Neg** | **Neg** | **NA** | **NA** | **Neg** | **Pos** |
| 4933 | LAIV/COM | **NA** | **NA** | **Neg** | **Pos** | **Neg** | **Pos** | **Neg** | **Pos** | **Neg** | **Pos** |
| 5182 | LAIV/COM | **NA** | **NA** | **Neg** | **Neg** | **NA** | **NA** | **NA** | **NA** | **Neg** | **Neg** |
| 5185 | LAIV/COM | **NA** | **NA** | **Neg** | **Neg** | **Neg** | **Neg** | **Neg** | **Pos** | **Pos** | **Pos** |
| 5174 | NO VAC/CHA | **Pos** | **Pos** | **Pos** | **Pos** | **Pos** | **Pos** | **Neg** | **Pos** | **Pos** | **Pos** |
| 5179 | NO VAC/CHA | **Neg** | **Pos** | **Neg** | **Pos** | **Neg** | **Pos** | **Neg** | **Pos** | **Pos** | **Pos** |
| 4553 | NO VAC/CHA | **NA** | **NA** | **NA** | **NA** | **NA** | **NA** | **NA** | **NA** | **NA** | **NA** |
| 5192 | NO VAC/CHA | **NA** | **NA** | **NA** | **NA** | **NA** | **NA** | **NA** | **NA** | **NA** | **NA** |
| 4495 | NO VAC/CHA | **Neg** | **Neg** | **Pos** | **Neg** | **Pos** | **Neg** | **Neg** | **Neg** | **Pos** | **Neg** |
| 4944 | NO VAC/CHA | **NA** | **NA** | **NA** | **NA** | **Neg** | **Neg** | **NA** | **NA** | **Neg** | **Neg** |
| 4490 | NO VAC/CHA | **Neg** | **Neg** | **Pos** | **Pos** | **Pos** | **Pos** | **Pos** | **Pos** | **Pos** | **Pos** |
| 5167 | NO VAC/CHA | **Neg** | **Pos** | **Neg** | **Pos** | **Neg** | **Pos** | **Neg** | **Pos** | **Pos** | **Pos** |
| 4493 | NO VAC/CHA | **NA** | **NA** | **Neg** | **Neg** | **Neg** | **Neg** | **Neg** | **Neg** | **Pos** | **Neg** |
| 4938 | NO VAC/CHA | **NA** | **NA** | **NA** | **NA** | **NA** | **NA** | **NA** | **NA** | **Pos** | **Neg** |

^a^ Abbreviations: dpc: days post-contact; NA: not applicable; Neg: negative; Pos: positive

^b^ Only the samples which obtained full length H1and/or H3 sequences are considered as positive.

^c^ Samples with Ct values above 38 tested by matrix RRT-PCR are considered as not applicable (NA) samples and are not submit for next generation sequencing.
