# Supplementary figures and images for: Exploring heterologous prime-boost vaccination approaches to enhance influenza control in pigs
Source: Vet Res. 2020 Jul 9;51:89. doi: 10.1186/s13567-020-00810-z (PMC7344353; doi:10.1186/s13567-020-00810-z)

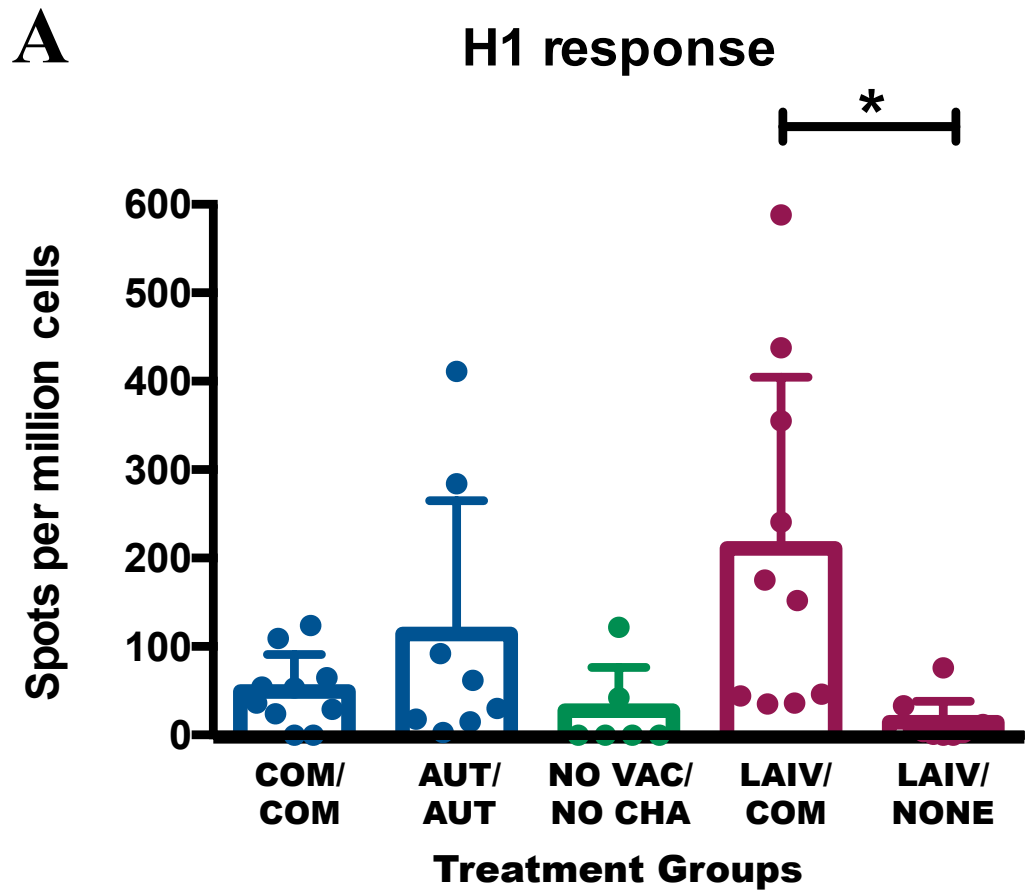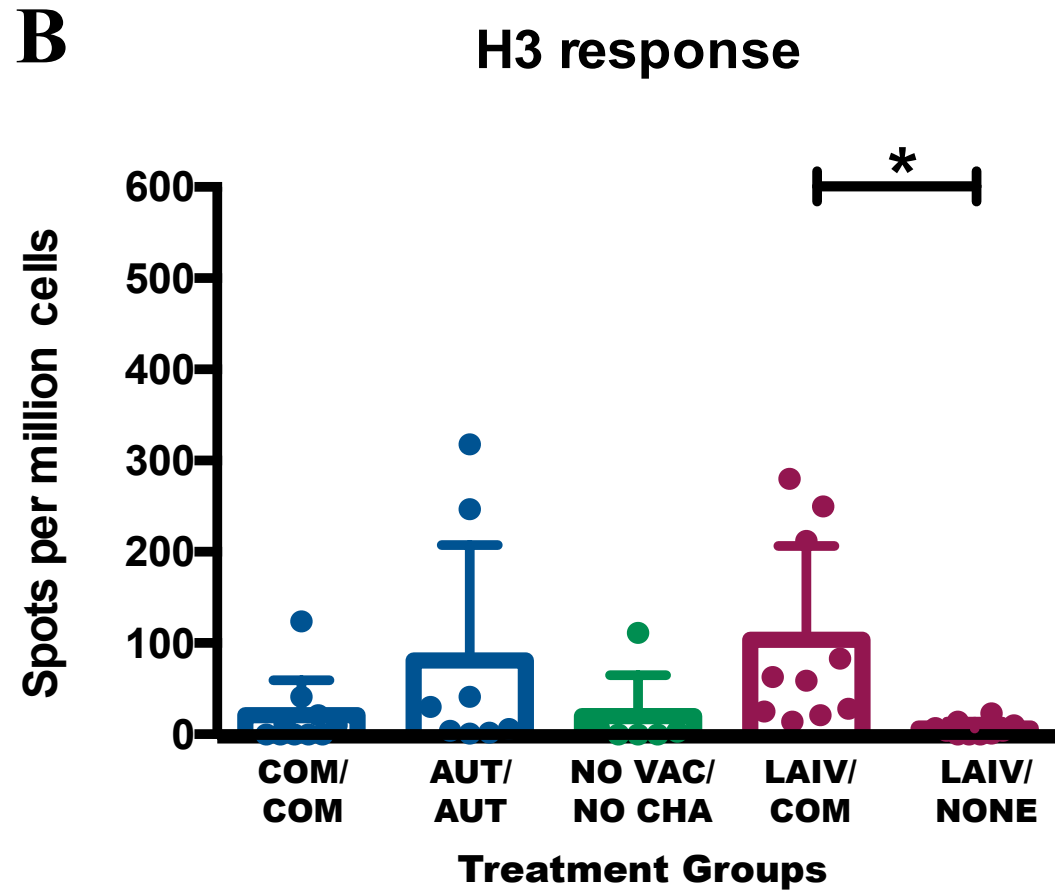

Supplement: Supplementary file 5 — Additional file 5. The number of IFN-γ specific H1 (A) and H3 (B) secreting cells in pigs from peripheral blood mononuclear cells (PBMC) collected at necropsy by treatment group. The number of IFN-γ secreting cells in each treatment group are summarized as mean ± SD. The spot counts for individual pigs are displayed as data points. The asterisks denote the significant difference (P < 0.05) of the number of IFN-γ secreting cell spots between groups. The dark blue, green and dark red bars and/or data points represent the pigs from the whole inactivate vaccine comparisons, negative control and live attenuate vaccine comparisons, respectively. [file 13567_2020_810_MOESM5_ESM.pdf]
